# Employing a single trial motor equivalent analysis for the assessment of motor learning

**DOI:** 10.1007/s00221-025-07123-7

**Published:** 2025-06-20

**Authors:** Matthew Beerse, Kimberly E. Bigelow, Joaquin A. Barrios

**Affiliations:** 1 Department of Health and Sport Science, University of Dayton, 300 College Park, Dayton, OH 45469-2968 USA; 2 Department of Mechanical and Aerospace Engineering, University of Dayton, Dayton, OH USA; 3 Department of Physical Therapy, University of Dayton, Dayton, OH USA

**Keywords:** Uncontrolled manifold, Motor learning, Methodology, Variability

## Abstract

The uncontrolled manifold analysis (UCM) is a useful technique for motor learning research enabling the classification of movement variability into solutions and errors. Less explored methodological considerations within the UCM framework are the selection of mean configurations outside of the current performance, as found in the Motor Equivalence Analysis, and a single trial approach. In this study, we demonstrated how calculating deviations away from varying mean configurations within the UCM influences the results and interpretations within motor learning experiments. Twelve young adult subjects (9F/3 M, 20.53 ± 1.25 years old) practiced the kettlebell swing over a one-week time period. We compared deviations from the mean configuration across all repetitions, to the mean of the first ten repetitions before practice and to the mean of their last ten repetitions after practice. Results suggested that subjects abandoned their initial mean performance within the first sets of kettlebell swings and reduced their errors and solutions towards what would become their mean performance after practice. They continued to refine their performance 1 week later. Subjects then completed a transfer task, testing their ability to adapt to a water-filled kettlebell. We evaluated deviations from their mean performance with the metal kettlebell and their mean performance with the water-filled kettlebell. Subjects did not reduce errors towards their mean metal kettlebell performance, but instead towards a new performance that matched the dynamics of the water-filled kettlebell. When performance is expected to change, i.e., motor learning, assessing how the variance structure changes with respect to different mean configurations can provide further insight when using a UCM approach.

## Introduction

The uncontrolled manifold (UCM) analysis partitions variability of local-level parameters, such as segment angles (Wu et al. 2009) or joint torques (Yen et al. 2009), into one of two subspaces dependent on whether or not that variability changes a task-level goal, such as center of mass (COM) position or vertical ground reaction force. The implementation of the UCM analysis was imperative for the motor control insight of abundance, suggesting that the neural system exploits variability at a local-level to stabilize performance at a task-level (Scholz and Schöner 1999; Latash et al. 2005). In other words, not all motor variability should be considered error, but instead some variability is a hallmark of a skilled performer. Since its introduction, the UCM analysis has been employed to test questions of motor control (Toney and Chang 2016; Beerse and Wu 2019), identify which task-specific performance variables are stabilized by the nervous system (Yamagata 2021; Bennett et al. 2024), evaluate clinical populations (Toney-Bolger and Chang 2022; Ibara et al. 2023), and assess variability during motor learning (Kang et al. 2004; Yang and Scholz 2005).

When conducting a UCM analysis, a task-level variable must be identified, which is assumed to represent the motor behavior goal (Scholz and Schöner 1999). This task-level variable generally takes two forms, dependent on the whether the motor task seeks to maintain a static state or is cyclical. Within-trial variables are implemented for tasks whose goal is to maintain an average or cued performance, such as a consistent COM position during quiet stance (Scholz et al. 2012) or matching a cued total force during a finger pressing task (Mattos 2015; de Freitas and Latash 2019; Grover et al. 2022). Between-trial variables are employed for cyclical tasks, most often the mean configuration of the local-level variables across cycles, such as segment angles during walking (Shafizadegan et al. 2022) or joint torques during hopping (Yen et al. 2009). The mean configuration as the goal of the motor behavior works well for practiced motor skills, as first outlined by Scholz and Schoner (1999), however, this expectation is less valid for motor learning paradigms, where the individual updates their motor behavior goal as part of the learning process (Scholz et al. 2003; Yang et al. 2007; Wu et al. 2012). For novel static tasks, this complication has been addressed by assessing shorter trials to reduce the chance of capturing learning effects (Wu et al. 2012, 2015). For cyclical tasks, calculating the mean configuration from a subset of cycles, rather than all cycles, has been used in adaptation research (Scholz et al. 2007; Mattos et al. 2011; Mattos 2015; Selgrade and Chang 2015), which could be similarly applied to motor learning paradigms.

A variation of the UCM analysis, termed the Motor Equivalence Analysis, was first introduced by Scholz et al., (2007) when evaluating COM control (task-level variable) through joint angle variability (local-level variable) during quiet standing with a surface perturbation. The mean configuration employed was the average joint angles before the perturbation. Deviations of the joint angles during the perturbation were then compared to the mean configuration and projected onto motor equivalent (ME), i.e., solutions, and non-motor equivalent (nME), i.e., errors, subspaces. This analysis has since been used to further define changes of variance structure to perturbations or when making quick actions (Yang et al. 2007; Mattos 2015; Ambike et al. 2017; de Freitas and Latash 2019). Additionally, the Motor Equivalence Analysis has been implemented to compare across repetitions, which is a common level of analysis for motor learning. Mattos et al. (2011) assessed how individuals adapted to a perturbation during reaching, where the mean configuration was the average joint angles from the unperturbed repetitions. Building upon this approach, Selgrade and Chang (2015) calculated deviations during single-leg hopping when adapting joint torque variability to a cued force. The mean configuration was taken from the last thirty repetitions of the trial, as this was expected to represent the motor behavior the subjects were actively modifying towards. During motor learning, one can similarly view the intended motor behavior goal as not being represented by current performance, but instead as dynamically updating (Pacheco et al. 2019). Therefore, employing mean configurations from early or later performances has the potential to provide insight to how individuals move towards or away from different solutions with practice.

Another benefit for motor learning research within the Motor Equivalence Analysis is the capacity to evaluate shorter timescales, in other words repetition-to-repetition changes rather than summed variance over many repetitions (Selgrade and Chang 2015). Abrupt performance adjustments from one repetition to the next have been found in other motor learning studies, where subjects demonstrate a “discontinuous jump” to a new performance and set of solutions (Liu and Newell 2015; Pacheco et al. 2020), which will likely be missed if summing variance across repetitions (Sternad 2018; Pacheco et al. 2019). A “single-trial” UCM analysis was first introduced for the evaluation of a finger force ramping task (Scholz et al. 2003). Rather than across trials, the UCM analysis was conducted on a set of time samples within the trial. For cyclical motor skills, this approach was adapted to the single cycle deviation analysis, which projects the deviations from the mean configuration of individual repetitions rather than summing variances across repetitions (Selgrade and Chang 2015). This is not to say that the summed variance approach is unsuitable for motor learning, as most implementations of the UCM analysis to date have taken this approach (Domkin et al. 2002, 2005; Kang et al. 2004; Yang and Scholz 2005; Beerse et al. 2020). Indeed, these larger time scales have been insightful for motor learning research demonstrating a general pattern that individuals initially reduce errors with practice, followed by a reduction of solution variability with further practice (Latash et al. 2005). Moreover, practice specifically designed with high variability and increasing complexity can increase solution variability (Wu et al. 2012). Instead, we propose that distinct motor learning questions can be addressed at a repetition-by-repetition timescale, an important quality for motor learning research (Newell and Liu 2020).

In this study, we evaluated how error and solution deviations evolved over a one-week period when novices practiced the kettlebell swing. We employed three mean configurations from which the deviations were calculated: (1) the mean segment angles across all cycles, termed traditional as this follows the traditional UCM approach; (2) the mean segment angles of the first ten repetitions performed, termed initial; (3) the mean segment angles of the last ten repetitions performed, termed practiced. We hypothesized that the subjects would abandon their initial configuration within the first sets of the kettlebell swing, as represented by an increase of nME deviations, i.e., errors. In addition, we expected that subjects would shift towards their practiced configuration with practice represented by a reduction of nME deviations followed by a reduction of ME deviations, i.e., solutions. Subjects then completed a transfer test by performing repetitions with a water-filled kettlebell. We compared two mean configurations: (1) the mean segment angles with their practiced metal kettlebell after one week of practice and (2) the mean segment angles for the water-filled kettlebell. We hypothesized that subjects would reduce their nME deviations for the water-filled kettlebell mean configuration but not the metal kettlebell mean configuration, suggesting a new intended motor behavior goal that better matches the dynamics of the water-filled kettlebell.

## Methods

### Participants

Twelve young adults (9F/3 M, 20.53 (1.25) years, 1.69 (0.08) m, 62.3 (11.4) kg) participated. Inclusion criteria included participation in resistance training for at least six months at a frequency of ≥ 2 days/week and that the kettlebell swing was a novel movement. Exclusion criteria included neurological, muscular or cognitive disorders, and current or previous injuries, particularly of the lower back, which could be made worse by exercise. In addition, subjects were excluded if they had previously been coached on the kettlebell swing or attempted to learn the movement independently. This study was approved by the institutional review board at the hosting university. Informed consent was obtained from all individual participants included in the study.

### Protocol

We collected kinematic data using an 8-camera Vicon motion capture system (Centennial, CO, USA) at a sample frequency of 100 Hz. A full-body PSIS marker set was attached to each subject according to the Vicon Plug-In Gait model (Gutierrez-Farewik et al. 2006). An extra marker was placed on the side of the kettlebell to identify repetitions. Kinematic data was filtered using a fourth-order zero-lag Butterworth filter with a 6 Hz cutoff frequency.

Subjects performed kettlebell swings on five separate days over the course of a one-week period (Table 1). We collected data on the subjects’ kettlebell swing performance on the first and last day. During the five days in between, subjects practiced the kettlebell swing on three of those days with supervision from researchers. The subjects did not practice on the other two days. Subjects with a body mass equal to or greater than 165 lbs used a 25 lbs kettlebell and subjects with less than a body mass of 165 lbs used a 15 lb kettlebell. The selection of this body mass cut-off and kettlebell weight was informed by previous intervention studies focused on exercise (Lake and Lauder 2012; Fortner et al. 2014). Since our goal was to learn the skill and not exercise, we reduced the weight used in these studies by 5 lbs to minimize the chance of fatigue. In a previous study that used the same sets, repetitions, and load cut-offs, the maximum rating of perceived exertion was 4 out of 10 suggesting that fatigue was minimized (Beerse et al. 2020).

**Table 1:** Overview of the data collection conditions and practice session

Day 1			Days 2–6			Day 7	
3 sets of 20 repetitions	5 sets of 20 repetitions	3 sets of 20 repetitions	5 sets of 20 repetitions	5 sets of 20 repetitions	5 sets of 20 repetitions	3 sets of 20 repetitions	3 sets of 20 repetitions with water-filled kettlebell
*Pre-practice*	Practice	*First practice*	Practice			*Post-practice*	*Adaptation*

Italics indicates a condition where data was collected

Kinematic data were collected at four time points during the study. On the first day, three sets of 20 repetitions were collected before practice (*pre-practice)* and three sets of 20 repetitions were collected after a practice session (*first practice*). All practice sessions comprised of five sets of 20 repetitions. One week later, we collected data on three sets of 20 repetitions (*post-practice*). As previously mentioned, within that week subjects completed three practice sessions on three separate days. An *adaptation* condition immediately followed the *post-practice* sets, where subjects performed three sets of 20 repetitions with a water-filled kettlebell. The water-filled kettlebell was equivalent in mass as their practiced kettlebell, but larger in size (diameter: practiced = 14 cm; water = 32 cm). Subjects rested for a minimum of three minutes in between each set of kettlebell swings to minimize fatigue.

Before the first *pre-practice* set of kettlebell swings, subjects watched a video of reconstructed motion capture data of a skilled demonstrator and listened to a list of verbal cues, such as “push the hips back, keeping the back straight”. These forms of instruction were available to the subjects before each set and followed a learner-regulated approach, where subjects selected when and how often they wanted to rewatch the video and hear the cues. Subjects opted to receive these instructions on average 3.9 times (range: 2–8), not including the required viewing at the beginning of the study. The minimum possible opportunities to receive instructions was 26, once before each new set. Subjects received no augmented feedback during the data collection or practice sessions.

### Data analysis

Kettlebell swing repetitions were partitioned using the marker on the right side of the kettlebell. The first and last repetition of each set were removed from analysis, as these repetitions often differ from the rest of the repetitions even for skilled performers (Bullock et al. 2017). The task-level variable within the UCM analysis was a consistent vertical COM trajectory across repetitions and the local variables were segment angles. Our previous findings suggested that subjects stabilized vertical COM position with segmental angle variability when learning the kettlebell swing (Beerse et al. 2020). Seventeen sagittal plane segment angles were calculated: foot, shank, thigh, pelvis, upper arm, lower arm, and hand bilaterally, and the trunk, head, and kettlebell.

We conducted a Motor Equivalence Analysis to generate error and solution deviations for each kettlebell swing repetition (Scholz et al. 2003; Mattos et al. 2011; Selgrade and Chang 2015). The segment angles were time normalized to every 1% of the kettlebell swing cycle. The following UCM analyses were conducted at each 1% and summed across these time points to generate single values for each repetition. For the evaluation of the *pre-practice*, *first practice*, and *post-practice* conditions we employed three different mean configurations. The traditional mean configuration was the average segment angles across all repetitions for that condition, making the assumption that subjects were attempting to stabilize an average posture throughout the sets (Scholz and Schöner 1999). The initial mean configuration was the average segment angles of the first ten repetitions of the *pre-practice* condition, making the assumption that subjects were attempting to stabilize their initial posture. The practiced mean configuration was the average segment angles of the last ten repetitions of the *post-practice* condition, making the assumption that subjects were attempting to stabilize what would eventually become their preferred posture.

Geometric models were constructed to relate changes of sagittal plane segment angles to vertical COM position and then linearized around each mean configuration to create the Jacobian (J). The null space of the Jacobian defines the uncontrolled space, or solution space, as defined in Eq. 1.

1 $$ J\left( {\theta^{^\circ } } \right) \cdot \varepsilon = 0 $$

Deviations of the segment angles (θ) away from the mean configuration (θ°) were calculated and were partitioned (Eqs. 2 and 3) into two subspaces, solutions (Θ_UCM_) or errors (θ_ORT_).

2 $$ \theta_{UCM} = \mathop \sum \limits_{i = 1}^{n - d} \varepsilon_{i}^{T} \cdot \left( {\theta - \theta^{^\circ } } \right) \cdot \varepsilon_{i} $$

3 $$ \theta_{ORT} = \left( {\theta - \theta^{^\circ } } \right) - \theta_{UCM} $$

At this point, rather than summing these deviations to variances, we normalized the deviations by the square root of their degrees of freedom (Eqs. 4 and 5).

4 $$ motor \; equivalent \; deviations = \frac{{\theta_{UCM} }}{{\sqrt {n - d} }} $$

5 $$ non{ - }motor \; equivalent \; deviations = \frac{{\theta_{ORT} }}{\sqrt d } $$

The motor equivalent deviations (ME) contained the solution space deviations, while the non-motor equivalent deviations (nME) contained the error space deviations. A ratio of the ME/nME deviations was calculated as a synergy index (Scholz et al. 2007; Mattos et al. 2011) and log transformed to account for a non-normal distribution.

We implemented a similar approach to evaluate the *adaptation* condition to the water-filled kettlebell. For this evaluation, we employed only two mean configurations, the traditional and the practiced. The same definitions for these mean configurations applied. Therefore, the traditional mean configuration assumed that subjects modified their deviations towards the average posture of the *adaptation* condition with the water-filled kettlebell. This approach expected the modified task dynamics of the water-filled kettlebell to demand the emergence of a new mean configuration that subjects sought to stabilize. The practiced mean configuration assumed that subjects modified their deviations back towards the posture they preferred with the metal kettlebell during the *post-practice* condition.

### Statistical analysis

To compare how subjects modified their deviations when learning the kettlebell swing, we calculated the mean ME, nME, and SCIDS for the first ten and last ten repetitions (labeled Start and End, respectively) for each condition (*pre-practice*, *first practice*, and *post-practice*) and mean configuration (traditional, initial, and practiced). For ME and nME deviations, differences between the mean configurations were not compared as it was expected that deviations were larger when using a mean configuration calculated from outside of the current performance. In other words, it was reasonable to expect that the traditional mean configuration would present with smaller deviations compared to the initial and practiced, except for the start of the *pre-practice* and end of the *post-practice* conditions, respectively. Therefore, we conducted two-way ANOVAs (3 condition × 2 time) with repeated measures on ME and nME deviations to compare differences from the start to the end of a condition (time factor) and between conditions (condition factor). Shapiro–Wilk tests were conducted to assess normality and a log transformation was applied to non-normal variables. Tukey post-hoc tests were conducted in the case of statistically significant main effects or interactions. For the ME/nME deviation ratio, we conducted a three-way ANOVA (3 condition × 2 time × 3 configuration) with repeated measures. The mean configuration was included as a factor since the ratio removes the inherent magnitude effect described earlier making it a more meaningful comparison.

To assess how subjects adapted to the water-filled kettlebell during the transfer task, we conducted paired t-tests on the traditional and practiced mean configurations, separately. Again, we chose not to include the mean configuration as a factor due to the expectation that the traditional mean configuration will result in smaller deviations. Whether subjects reduced their nME deviations during the *adaptation* condition was the more pertinent question. Eta squared and Cohen’s d were calculated as effect sizes. Statistical analysis was conducted in R Statistical Software (v4.3.2; R Core Team 2023). Significance level was set at alpha = 0.05.

## Results

### Learning the metal kettlebell

**Fig. 1:**
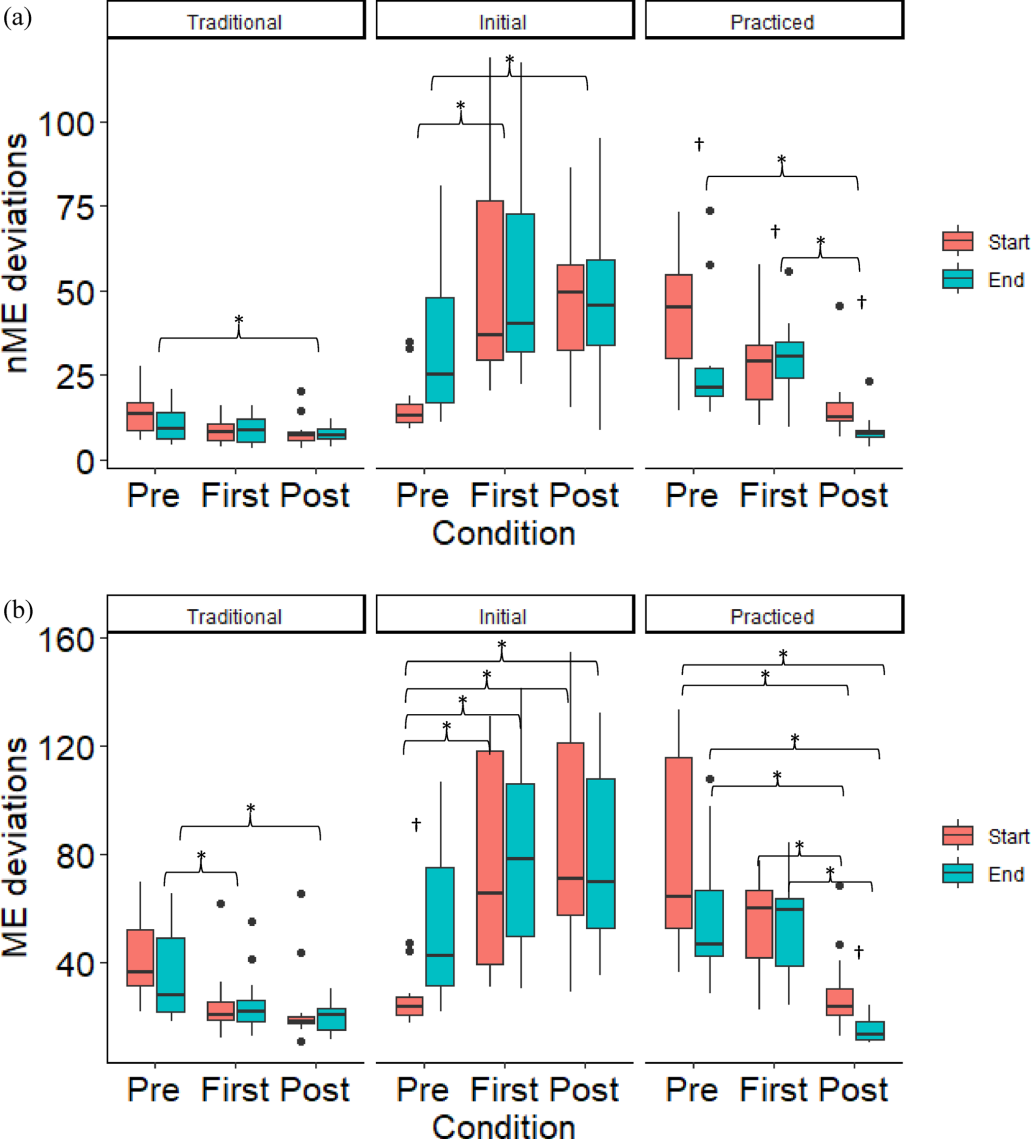

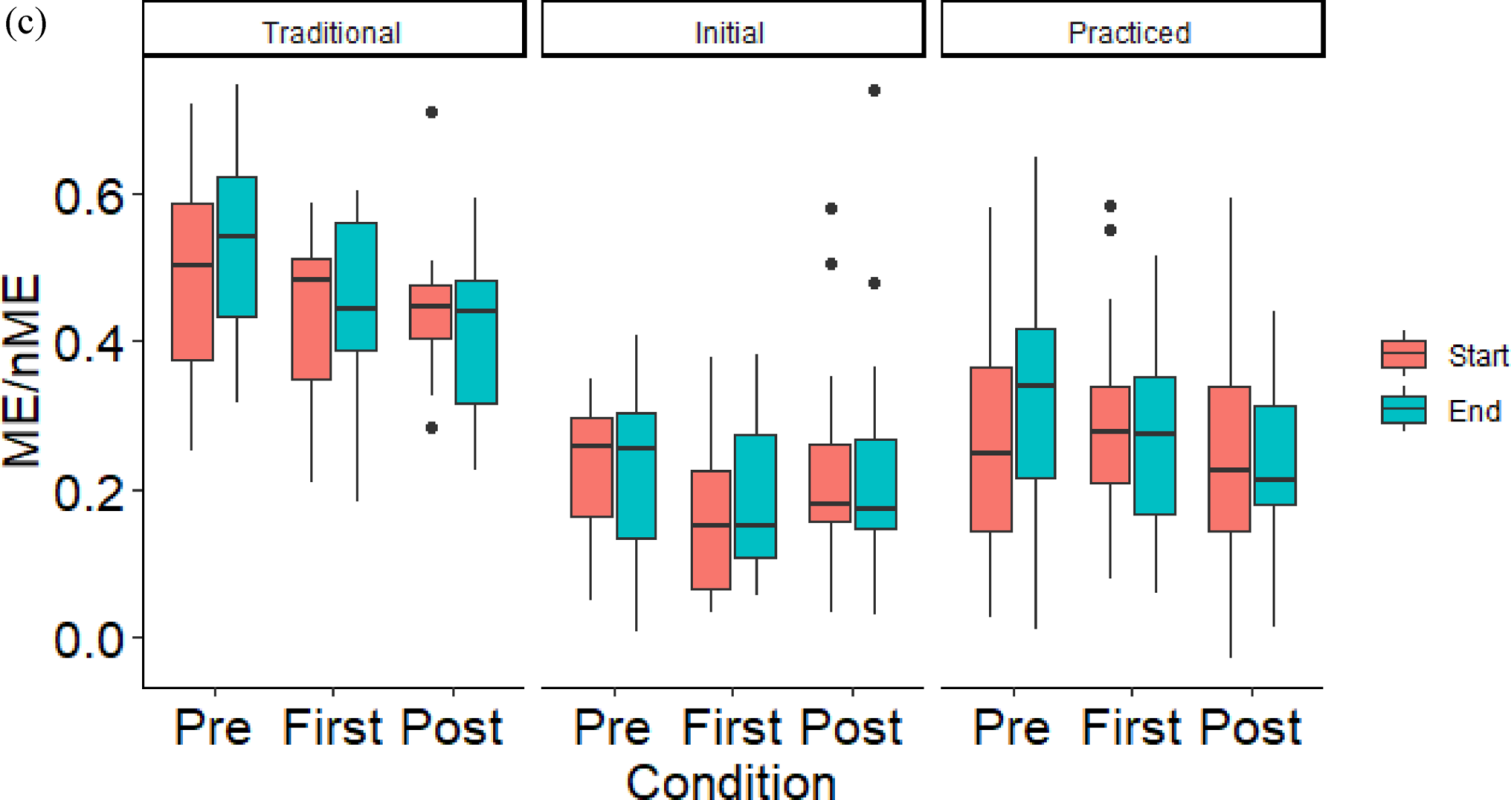
Mean Motor Equivalence Analysis variables of nME deviations (**a**), ME deviations (**b**), and the log transformed ratio of ME/nME deviations (**c**) of the first (Start) and last (End) ten repetitions for the *pre-practice*, *first practice*, and *post-practice* conditions, separated by mean configuration. The nME deviations correspond to segment angle variability that changes the vertical center-of-mass trajectory across repetitions, while ME deviations correspond to variability that does not. Therefore, nME deviations are interpreted as error variability and ME deviations are interpreted as solution variability. *Indicates a significant condition effect and † indicates a significant time effect

For the traditional and practiced mean configurations, the nME deviations tended to decrease with practice, while they increased for the initial mean configuration (Fig. 1a). A condition main effect was found for the traditional mean configuration (F(2,66) = 4.347, *p* = 0.017, η^2^ = 0.116), where nME deviations decreased from the *pre-practice* condition to the *post-practice* condition (*p* = 0.018). There was a condition main effect (F(2,66) = 32.810, *p* < 0.001, η^2^ = 0.499) and a time main effect (F(1,66) = 7.635, *p* = 0.007, η^2^ = 0.104) for the practiced mean configuration. The *post-practice* condition was less than both the *pre-practice* (*p* < 0.001) and the *first practice* (*p* < 0.001) conditions. The nME deviations decreased from the start to the end of the conditions (*p* = 0.007). For the initial mean configuration, there was a condition main effect (F(2,66) = 12.927, *p* < 0.001, η^2^ = 0.281). The *pre-practice* nME deviations were smaller compared to the *first practice* (*p* < 0.001) and the *post-practice* (*p* < 0.001) conditions.

The ME deviations tended to decrease for the traditional and practiced mean configurations, but increased for the initial mean configuration (Fig. 1b). A significant condition main effect was found for the traditional mean configuration (F(2,66) = 12.282, *p* < 0.001, η^2^ = 0.271), where the ME deviations were greater during the *pre-practice* condition compared to the *first practice* (*p* = 0.001) and the *post-practice* condition (*p* < 0.001). For the practiced mean configuration, a condition by time interaction was found (F(2,66) = 3.378, *p* = 0.040, η^2^ = 0.093). The start and end of the *post-practice* condition had smaller ME deviations compared to all other time points (all *p*-values less than *p* = 0.002). The end of the *post-practice* condition had smaller ME deviations compared to the start of the *post-practice* condition (*p* = 0.009). There was also a significant condition by time interaction for the initial mean configuration (F(2,66) = 3.155, *p* = 0.049, η^2^ = 0.087), where the start of the *pre-practice* condition had smaller ME deviations compared to all other time points (*p* < 0.001) including the end of the *pre-practice* condition (*p* = 0.024).

The traditional mean configuration ME/nME deviation ratio was greater compared to the initial and practiced mean configurations (Fig. 1c). There was a configuration main effect (F(2,198) = 59.663, *p* < 0.001, η^2^ = 0.376).

### Water-filled kettlebell transfer test

**Fig. 2:**
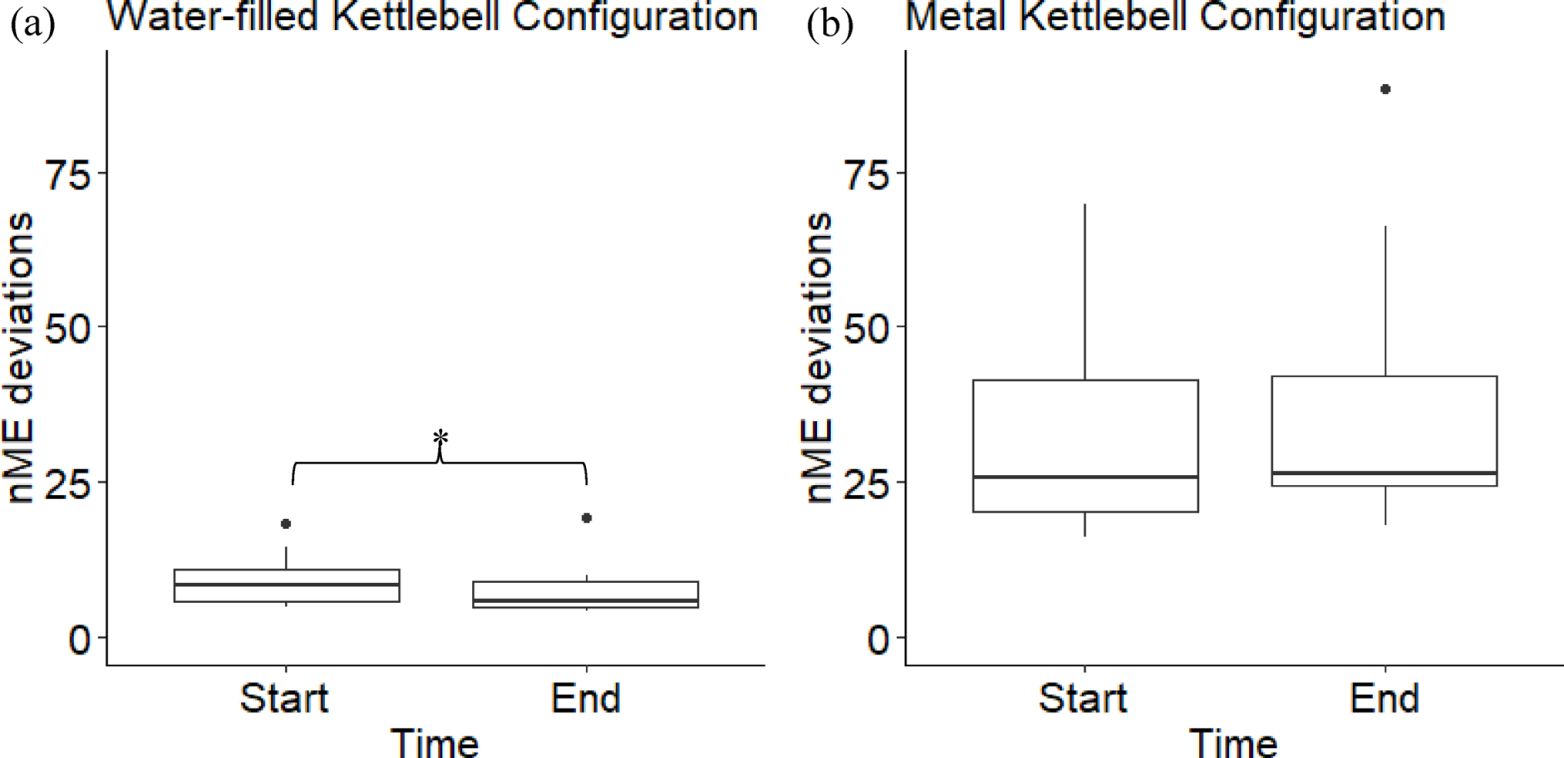
Box plots of the nME deviations, i.e., errors, of the first (Start) and last (End) ten repetitions of the *adaptation* condition for the mean configuration using the average segment angles when performing repetitions with the water-filled kettlebell (**a**) and the average segment angles when performing repetitions with the metal kettlebell during the *post-practice* condition (**b**). *Indicates a significant difference

**Fig. 3:**
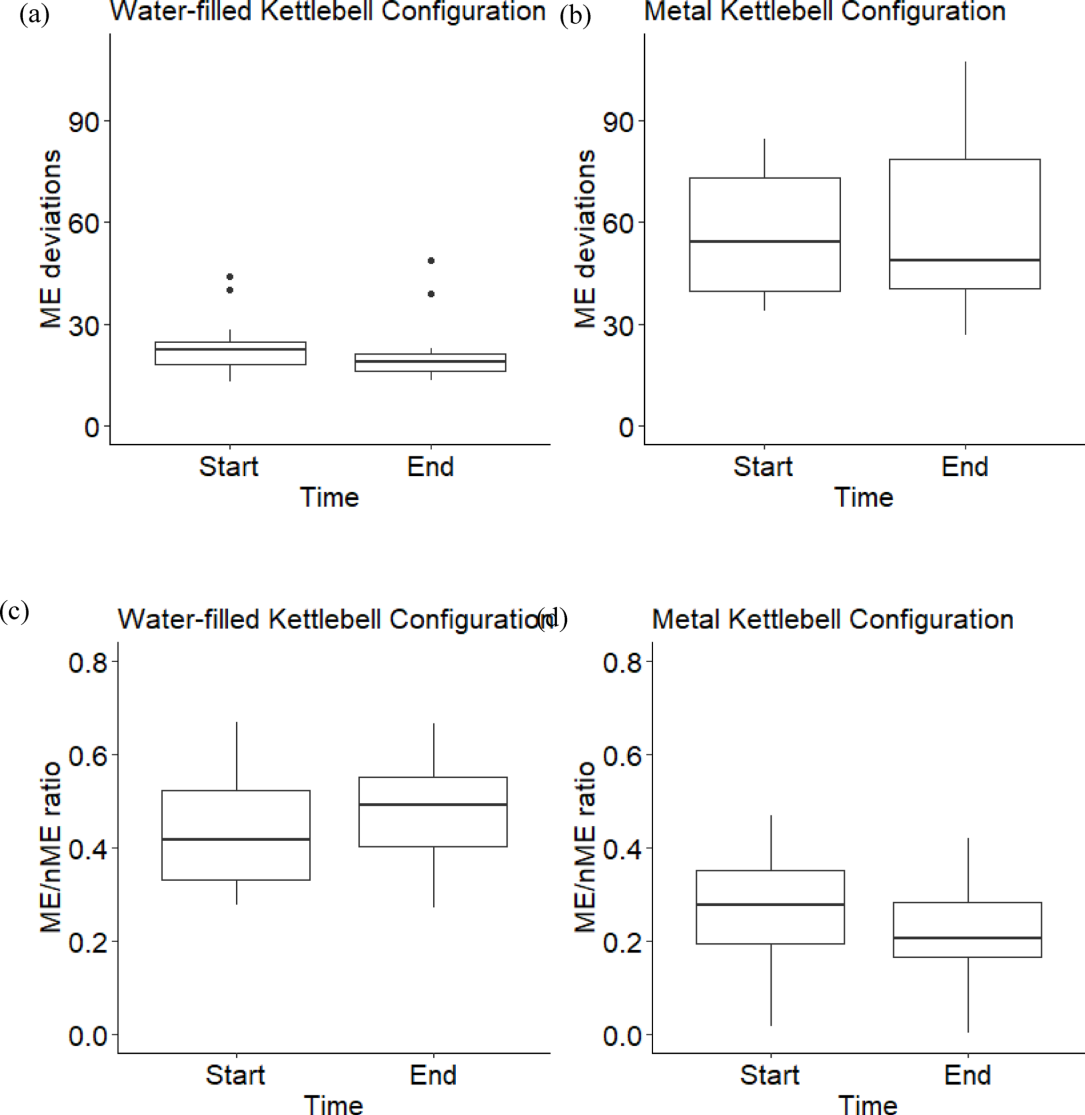
Box plots of ME deviations (**a**, **b**) and log transformed ME/nME deviation ratio (**c**, **d**) of the first (Start) and last (End) ten repetitions of the *adaptation* condition for the traditional and practiced reference configurations

On average, subjects adapted to the water-filled kettlebell by reducing errors towards mean segment angles that were distinct from their mean configuration with the metal kettlebell (Fig. 2). The average nME deviation at the end of the *adaptation* condition was significantly lower than the start for the traditional mean configuration (t(11) = 2.73, *p* = 0.020, d = 0.79) (Fig. 2a), but not the practiced mean configuration (t(11) = − 0.84, *p* = 0.418, d = − 0.24) (Fig. 3a, b) (Fig. 2b). There were no significant differences of ME deviations nor the ME/nME deviation ratio (Fig. 3).

## Discussion

In this study, we employed a single trial Motor Equivalence Analysis from the UCM framework to determine how local variability evolved over a one-week period when learning the kettlebell swing. Our first hypothesis was supported as subjects did not systematically reduce their deviations towards their initial configuration, but instead both nME and ME deviations increased as early as within the *pre-practice* condition. In partial support of our second hypothesis, subjects reduced nME deviations towards their practiced configuration as quickly as the *pre-practice* condition.

The traditional mean configuration employed in this study matches the original formulation of the UCM analysis (Scholz and Schöner 1999), which used a well-practiced motor skill and assumes that the average performance across the entire set of cycles best represents the central nervous system’s intended configuration. Caution about this approach for motor learning paradigms have been previously communicated (Scholz et al. 2003; Yang et al. 2007), as the intended configuration might be updated during performance as part of the motor learning process (Wu et al. 2012; Pacheco et al. 2019; Button et al. 2021). Assessing different mean configurations within a UCM framework has the potential to evaluate the intended configuration, that is likely dynamic during motor learning. Our results suggested that subjects abandoned their initial performance within the first day of practice as both ME and nME deviations increased during the *pre-practice* condition and to the *first practice* condition (Fig. 1a, b). These increased deviations do not follow the expected pattern, where variability tends to decrease with practice (Latash et al. 2005; Beerse et al. 2020). Likely, the increased deviations resulted from the initial mean configuration within the UCM model not matching the intended configuration. Here we demonstrate that modifying the mean configuration enables the assessment of the central nervous system’s intended configuration, somewhat analogous to the use of UCM to determine which task-level variables are stabilized during performance (Yamagata 2021; Bennett et al. 2024). The advantage of using UCM to compare mean configurations, rather than more traditional error measures, is the ability to collapse high dimensional data into variance structures (Rein 2012).

For the traditional mean configuration, our findings agreed with other motor learning studies using the UCM approach (Kang et al. 2004; Latash et al. 2005; Yang and Scholz 2005; Beerse et al. 2020), where nME and ME deviations decreased from the *pre-practice* condition to the *post-practice* condition (Fig. 1a). While similar results were found using the practiced mean configuration, two differences can be identified. First, the ME deviations did not decrease from the *pre-practice* condition to the *first practice* condition. The finding suggests that subjects did not identify what would become their preferred practiced solutions within the first day of practice. Instead, subjects sought to exploit the solutions of their current average performance, even though it was likely less desirable compared to the mean configuration they would ultimately adopt after the week of practice. This finding is similar to the concept of a local minima, where individuals adopt a “good enough” solution that might not be the most optimal (Pacheco et al. 2020). Of course, here we are assuming that the practiced configuration represents a more optimal solution.

The second difference was the presence of further refinement of variability during the *post-practice* condition, which was not captured with the traditional mean configuration. Specifically, both nME and ME deviations decreased from the *first practice* to the *post-practice* condition, as well as from the start to the end of the *post-practice* condition. Three days of practice over a one-week time period separated the *first practice* and *post-practice* conditions, therefore our results indicate the subjects continued to reduce their errors and prune their solutions during this practice. We postulate that the traditional mean configuration is insensitive to these changes due to the perpetuation of two motor goals, maintain steady-state performance while making adjustments towards more optimal solutions (Wu et al. 2014). Indeed, evidence suggests that individuals will seek to maintain their performance, while making adjustments towards new solutions (Liu and Newell 2015; Pacheco et al. 2020). It is unclear whether these new solutions were an attempt to match the subjects’ internal approximation of the “correct form” for the kettlebell swing or were seeking some other motor behavior goal such as maximizing the energy efficiency of their movement pattern (Wu et al. 2014; Selgrade and Chang 2015).

**Fig. 4:**
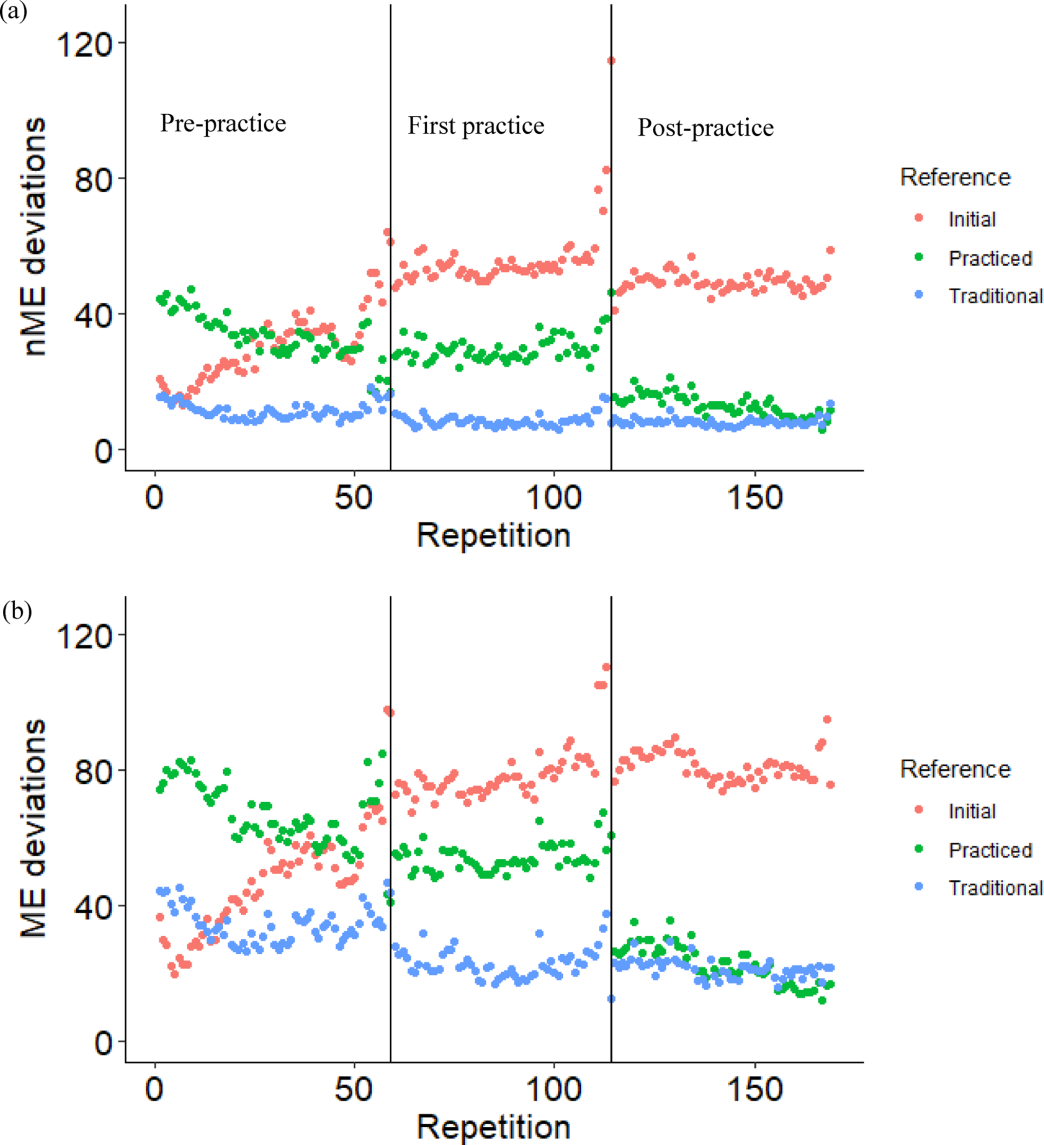

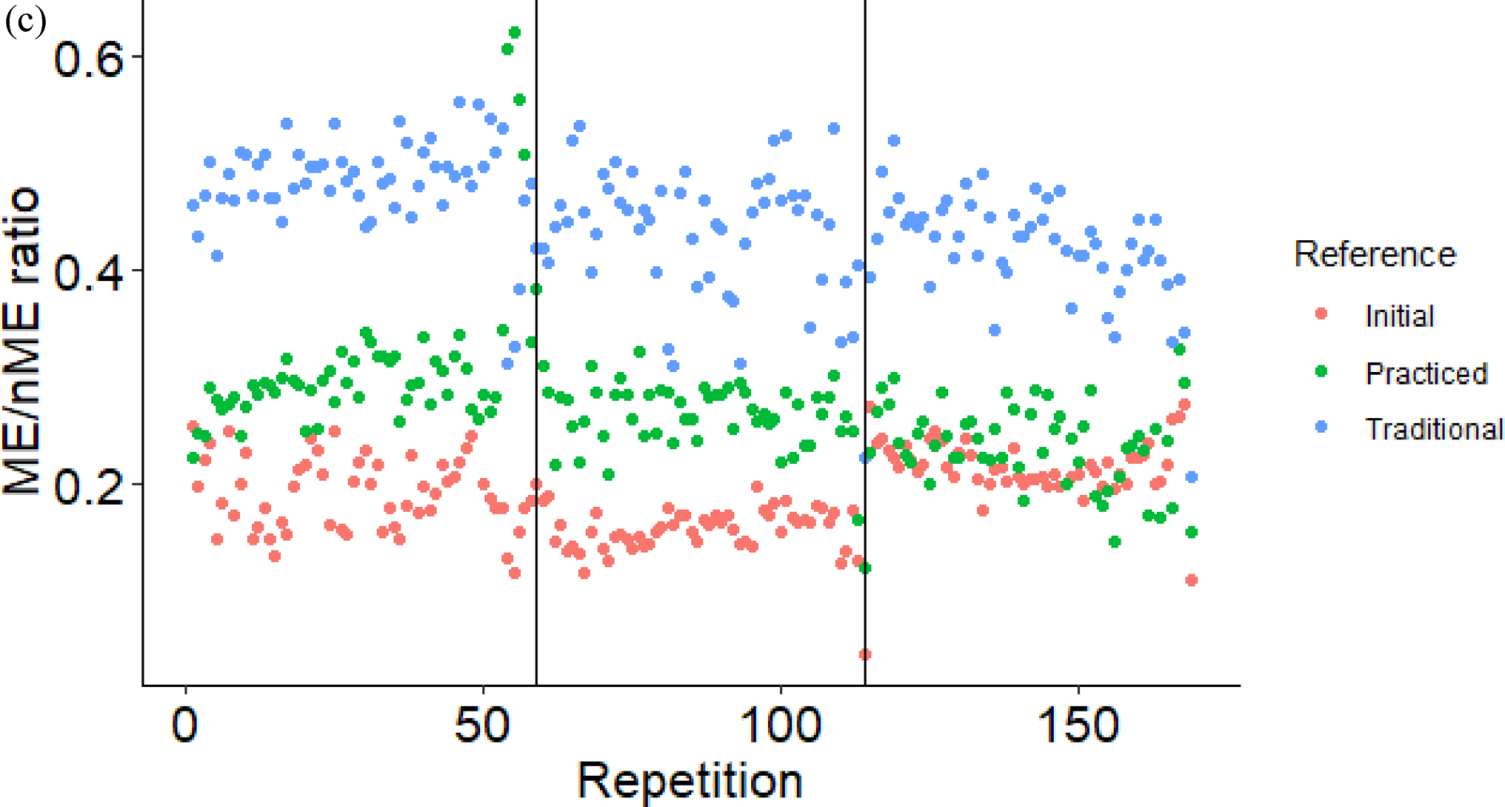
Ensemble averages of nME deviations (**a**), ME deviations (**b**), and the log normalized ME/nME deviation ratio (**c**) across the *pre-practice*, *first practice*, and *post-practice* conditions separated by each reference configuration. Vertical lines represent the separation of the end of a condition and the start of the proceeding condition

**Fig. 5:**
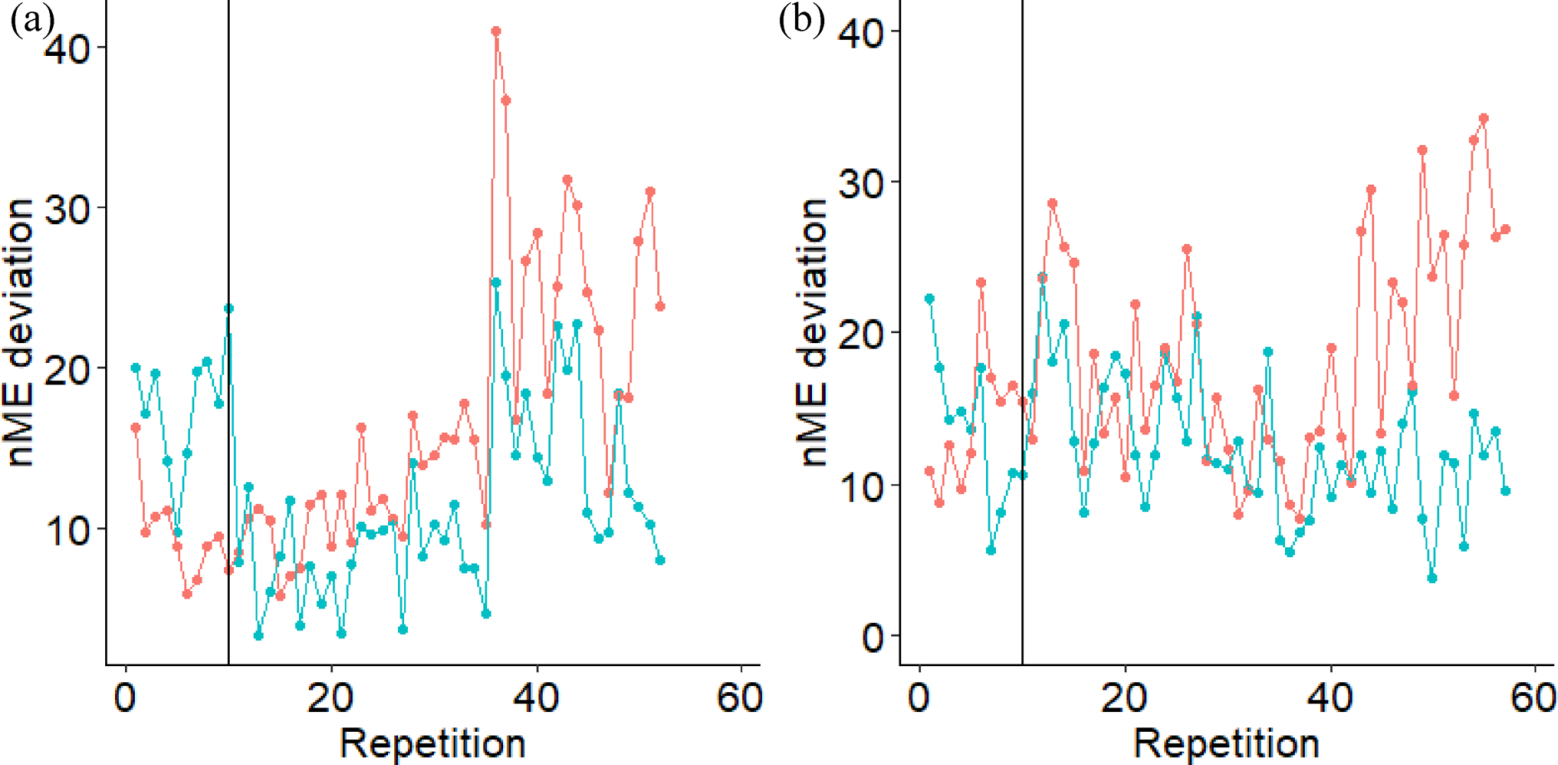
Example data for two individual subjects of nME deviations away from the initial mean configuration (red) and the traditional mean configuration (blue) during the *pre-practice* condition. The subject on the left (**a**) demonstrated a pattern where they reduced nME deviations around their initial mean configuration for a number of repetitions after the 10-repetition mark (solid vertical line). In contrast, the subject on the right (**b**) demonstrated a pattern where they did not reduce their nME deviations for the initial mean configuration even during the first ten repetitions

As potentially the first goal of motor learning, prioritization might have been given to stabilizing the current mean performance, based on the ME/nME deviation ratio. Across all time points, a greater ratio was found for the traditional mean configuration compared to the initial or practiced mean configurations. In other words, subjects better stabilized performance around their current mean configuration by operationalizing greater ME deviations relative to the nME deviations (Mattos et al. 2011; Mattos 2015). The evolution then of the nME deviations of each repetition across conditions highlights the continuous adjustments towards the practiced mean configuration (Fig. 4a). The nME deviations around the traditional mean configuration did not reduce from the start to the end of any single condition, while the nME deviations around the practiced mean configuration did, as well as from the *first practice* condition to the *post-practice* condition. It is important to note that the use of the practiced mean configuration taken from the end of the *post-practice* condition, insinuates that the central nervous system predetermined this intended configuration and then through practice the subjects learned how to stabilize this configuration. However, that is not an argument we are proposing. Instead, the change of nME and ME deviations suggest the timescale at which the subjects’ found their practiced mean configuration, as a function of the interaction between their intrinsic dynamics and the task demands (Pacheco et al. 2019; Newell and Liu 2020; Levin and Piscitelli 2022). For our study, it is evident that the practiced mean configuration did not fully emerge until after the first day of practice since both nME and ME deviations decreased from *first practice* to *post-practice*. Future applications of a single trial Motor Equivalence Analysis, as demonstrated here, could evaluate shorter timescales to better describe the evolution of the intended mean configuration. For example, mean configurations taken from the not-too-distant past from the current performance could provide evidence for how subjects update their intended mean configuration from prior attempts. The individual data plotted in Fig. 5 illustrates this smaller timescale evaluation as one subject (Fig. 5a) did reduce their nME deviations toward the initial mean configuration at the start of the *pre-practice* condition, while the other did not (Fig. 5b).

To assess performance during the transfer task of completing kettlebell swings with a water-filled kettlebell, we evaluated two mean configurations. Our hypothesis was supported, as subjects reduced their nME deviations towards their current mean performance, but not the mean configuration they employed for the metal kettlebell. At least in the short-term, subjects perceived their practiced configuration did not well match the task dynamics of the water-filled kettlebell, such as the momentum of the water and the difference in spatial dimensions. Comparing these two mean configurations within a Motor Equivalence Analysis provided insight to the intended configuration the individuals sought to stabilize with practice. This is further evidence for an emergent interpretation of motor learning (Liu and Newell 2015; Pacheco et al. 2019, 2020; Levin and Piscitelli 2022), as subjects had to respond to the modified task dynamics. Even if the subjects intended to match the mean configuration they learned for the metal kettlebell, they did not reduce their errors towards that configuration, at least within these first sets of attempts. Keeping in mind the potential prioritization to stabilize around the current mean performance, it is unclear whether with more practice subjects would have converged towards their metal kettlebell practiced mean configuration.

There are a number of limitations associated with this study. The first limitation is the selection of the first and last ten repetitions as the initial and practiced mean configurations, respectively. The selection of ten repetitions, while in some sense arbitrary, was hypothesis driven as we wanted to assess how these mean configurations may or may not have matched the intended configuration of the central nervous system. It was our expectation that each mean configuration did not match the intended configuration at each time point of the study. Another limitation was the possibility of conflating the results that were driven by the linearization of variability subspaces around a mean configuration that does not represent the average of the data. This is generally not a concern for the traditional mean configuration, since the average posture is utilized. To avoid conflation, we interpreted increased nME and ME deviations as the more likely result that the mean configuration did not match the intended configuration, rather than an intentional increase of errors or solutions, respectively. This is plausible because prior evidence of motor learning suggests that these errors and solutions generally decrease with practice and proficiency (Latash et al. 2005; Yang and Scholz 2005; Beerse et al. 2020). Therefore, it is important that future research intentionally test mean configurations within theoretical frameworks in order to avoid overstating potential statistical artifacts.

In conclusion, we demonstrated the utility of assessing different mean configurations, within a single trial UCM analysis for motor learning research following a Motor Equivalence Analysis approach. This approach has the potential to evaluate how subjects move away from or towards different intended configurations. Further, the cycle-by-cycle deviations provides higher resolution into the process of motor learning that is potentially overlooked with variances. In our study, individuals abandoned their initial configuration during the *pre-practice* condition and continued to refine their errors and solutions after one-week of practice. Moreover, when performing a transfer test with a water-filled kettlebell, subjects adopted a new configuration to match the altered dynamics of the task rather than attempting to adapt back to the practiced configuration they employed with the metal kettlebell.

## Data Availability

The data that support the findings of this study are available from the authors upon reasonable request.
